# The Influence of Timing of Surgery on Postoperative Length of Hospital Stay in Closed Ankle Fractures

**DOI:** 10.7759/cureus.73692

**Published:** 2024-11-14

**Authors:** Muhammad Mannan, Ali Ullah Ghauri, Faisal Karim, Usman Hafeez, Sarmad Khalil

**Affiliations:** 1 Trauma and Orthopaedics, Ghurki Trust Teaching Hospital, Lahore, PAK; 2 Trauma and Orthopaedics, Sheikh Zayed Medical College and Hospital, Rahim Yar Khan, PAK; 3 Orthopaedic Surgery, University Hospital Birmingham, Birmingham, GBR; 4 Trauma and Orthopaedics, University Hospitals Birmingham NHS Foundation Trust, Birmingham, GBR; 5 Trauma and Orthopaedics, Benzair Bhutto Hospital (BBH), Rawalpindi, PAK; 6 Trauma and Orthopaedics, University Hospital Birmingham, Birmingham, GBR; 7 Trauma and Orthopaedics, University Hospital Birmingham NHS Foundation Trust, Birmingham, GBR; 8 Trauma and Orthopaedics, Queen Elizabeth Hospital Birmingham, Birmingham, GBR

**Keywords:** ankle fractures, early presentation, hospital stay, late presentation, surgical timing

## Abstract

Background

Ankle fractures are prevalent lower limb injuries that often necessitate surgical intervention to restore joint stability and functionality. Delays in surgical management can extend hospital stays and elevate the risk of complications. This study aims to evaluate the effect of surgical timing on the postoperative length of hospital stay in patients with closed ankle fractures managed through open reduction and internal fixation (ORIF).

Objective

The objective of this study was to determine whether early surgical intervention (within eight hours of injury) reduces the mean postoperative length of hospital stay compared to delayed surgery (after eight hours) in patients with closed ankle fractures undergoing open reduction and internal fixation (ORIF).

Methods

A retrospective cohort study was conducted over six months, from February 26, 2021, to August 26, 2021, involving 80 patients with closed ankle fractures treated at the Orthopaedic Surgery Department of Benazir Bhutto Hospital, Rawalpindi. Patients were categorized into two cohorts based on the time elapsed between injury and surgical intervention: early surgery (≤8 hours, n=43) and late surgery (>8 hours, n=37). The primary outcome measured was the length of hospital stay following ORIF. Data were analyzed using the Statistical Package for the Social Sciences (SPSS) version 23 (IBM Corp., Armonk, NY. US). Independent sample t-tests, chi-square tests, and analysis of variance (ANOVA) were employed to assess differences between cohorts, with a p-value of less than 0.05 considered statistically significant.

Results

The mean age of the patients was 43.90 ± 14.64 years, comprising 58 (72.5%) males and 22 (27.5%) females. The overall mean length of hospital stay was 2.67 ± 0.87 days. Patients who underwent late surgery had a significantly longer hospital stay (mean 3.04 ± 0.80 days, n=37) compared to those treated early (mean 2.36 ± 0.82 days, n=43), with a p-value of <0.001. The stratified analysis revealed similar trends across various subgroups, including age and gender.

Conclusion

This retrospective cohort study concludes that delayed surgical management of closed ankle fractures is associated with a longer hospital stay compared to early intervention. These findings advocate for prompt surgical treatment to enhance patient outcomes by reducing postoperative hospitalization durations.

## Introduction

Ankle fractures are among the most common orthopedic injuries, accounting for approximately 7.5% of all fractures in adults. The estimated annual incidence is around 174 per 100,000 adults, with a bimodal distribution, primarily affecting younger males and older females due to varying mechanisms of injury [1]. These fractures frequently result from low-energy trauma, such as twisting injuries, but may also occur due to high-energy mechanisms, including falls from height or motor vehicle accidents [2]. The primary goal in managing ankle fractures is to restore the stability of the joint, achieve anatomical alignment, and prevent long-term complications such as post-traumatic arthritis [3]. The standard treatment for unstable ankle fractures is open reduction and internal fixation (ORIF), which ensures proper realignment and stabilization of the fractured bones [4].

The timing of surgical intervention plays a critical role in determining the overall outcomes for patients. Early surgical management, performed within 24 to 48 hours after the injury, has been associated with shorter hospital stays, fewer complications related to wound healing, and a lower risk of infection [5,6]. Early intervention allows for prompt fracture stabilization, minimizing soft tissue damage, and reducing the risk of complications from prolonged immobilization such as deep vein thrombosis (DVT) [7]. In contrast, surgery may be delayed due to factors such as swelling, the development of fracture blisters, or operating room availability. These delays can lead to extended hospital stays, slower recovery, and increased healthcare costs [8,9].

Numerous studies have explored the impact of surgical timing on outcomes in the management of ankle fractures. For example, Singh et al. reported that patients who underwent ORIF within 24 hours had significantly shorter postoperative hospital stays compared to those who had surgery later [8]. Similarly, Høiness and Strømsøe found that delayed surgery was associated with a higher incidence of wound infections and prolonged hospital stays in patients with closed ankle fractures [9]. However, other research has shown no significant effect of surgical timing on hospital stay or complication rates, highlighting the need for further studies to reach conclusive evidence [10].

In the specific context of closed ankle fractures, the influence of surgical timing remains a topic of ongoing debate, and there is limited data available at the local level. This study aims to bridge that gap by evaluating the impact of the timing of surgery - defined as early (within 8 hours from injury) versus late (more than 8 hours from injury) - on the length of postoperative hospital stays in patients undergoing ORIF. The expected findings will provide valuable insights to guide clinical decision-making and improve patient outcomes by optimizing the timing of surgical intervention.

## Materials and methods

Study design

This retrospective cohort study was conducted to evaluate the impact of surgical timing on the length of postoperative hospital stay in patients with closed ankle fractures treated with open reduction and internal fixation (ORIF) at the inpatient, outpatient, and emergency departments of the orthopedic surgery department at Benazir Bhutto Hospital, Rawalpindi, Pakistan, over a six-month period from February 26, 2021, to August 26, 2021. A total of 80 patients were included and categorized into two cohorts based on the time elapsed between injury and surgical intervention: early surgery (≤8 hours, n=43) and late surgery (>8 hours, n=37). The outcomes of these cohorts were compared to assess differences related to surgical timing.

The study received ethical approval from the hospital’s review board. Consecutive non-probability sampling was employed to include all eligible patients who met the inclusion and exclusion criteria during the study period, ensuring comprehensive case coverage. Data were retrospectively extracted from medical records, including demographic information, clinical variables, timing of surgery, and length of hospital stay.

Inclusion and Exclusion Criteria

Table 1 presents the inclusion and exclusion criteria.

**Table 1:** Inclusion and exclusion criteria

Inclusion Criteria	Exclusion Criteria
Patients aged 15 to 65 years	Open ankle fractures
Closed ankle fractures confirmed by clinical and radiographic examination	Unwilling/unable to provide consent
	Pre-existing infected skin, dirty wounds, or blisters at the fracture site

Data Collection Procedure

After obtaining ethical approval, eligible patients were recruited from the emergency, outpatient, and inpatient departments. Informed consent was obtained from all participants after thoroughly explaining the objectives of the study. Patients were divided into two groups based on the timing of surgical intervention: the Early Management Group, consisting of patients who underwent surgery within or equal to eight hours from the time of injury, and the Late Management Group, comprising those who underwent surgery more than eight hours after the injury. In all patients, ORIF was done with plates and screws for the fibula and distal tibia or only screw/K wire fixation for the medial malleolus. K wires for the medial bone pieces are repositioned and aligned after surgically exposing the bone and internally fixed with screws, plates, or K wires.

Demographic details, including age, gender, and place of residence, were recorded alongside clinical variables such as the time elapsed since injury and the length of hospital stay following ORIF. Reasons for surgical delays, including late presentation to the hospital, swelling at the fracture site, and unavailability of the operating theater, were also documented. All data were collected using a pre-designed proforma (Appendices) and a structured questionnaire to ensure consistency and accuracy in data collection.

Statistical analysis

The collected data were entered and analyzed using the Statistical Package for the Social Sciences (SPSS) version 23 (IBM Corp., Armonk, NY. US). While we did collect basic demographic and clinical data, comprehensive information on patient comorbidities, such as diabetes, hypertension, and cardiovascular diseases, was not systematically recorded or analyzed. Consequently, we did not perform a detailed assessment of how these comorbid conditions may have influenced the length of hospital stay. Qualitative variables, such as gender, place of residence, timing of surgery, and reasons for surgical delay, were expressed as frequencies and percentages. Quantitative variables, including age, time to surgery, and length of hospital stay, were summarized as mean values with standard deviations. Stratification was performed based on age, gender, and time to surgery to assess potential effect modifiers. Independent sample t-tests were employed to compare the mean length of hospital stay between the early surgery group (≤8 hours, n=43) and the late surgery group (>8 hours, n=37). For these t-tests, a critical t-value of approximately ±1.990 (based on 78 degrees of freedom) was considered statistically significant at a p-value of less than 0.05. Additionally, chi-square tests were utilized to analyze associations between categorical variables such as gender, presence of swelling, and operating theatre availability with the timing of surgery. Analysis of variance (ANOVA) was applied where relevant to compare means across multiple subgroups, including different age categories. Results were presented in tables for clarity and ease of interpretation.

## Results

In our study, 80 patients were included, and formal statistical analysis for sample size calculation was not performed; instead, consecutive non-probability sampling was utilized to include all available cases that met the inclusion and exclusion criteria within the designated timeframe. Of these, 58 (72.50%) patients were male and 22 (27.50%) patients were female. The male-to-female ratio of the patients was 2.6:1 with a mean age of 44.15 years (SD = 14.64), ranging from 17 to 65 years, reflecting a broad age range in the adult population. Regarding the timing of presentation, 37 patients (46.3%) presented late (more than 8 hours after injury) while 43 patients (53.8%) presented early (within 8 hours), indicating a nearly balanced distribution. Swelling at the fracture site was found in 42 patients (52.5%), which could contribute to delays in surgical intervention. Additionally, non-availability of the operation theater was reported in 16 cases (20%), potentially affecting the timeliness of surgery. These demographic and clinical characteristics provide an overview of our study population, establishing a foundation for analyzing the impact of these factors on outcomes such as the length of hospital stay. This is presented in Table 2.

**Table 2:** Distribution of patients

Category	Value
Total Patients	80
Mean Age (Years)	44.15
Age SD	14.64
Minimum Age	17
Maximum Age	65
Male Patients	58 (72.50%)
Female Patients	22 (27.50%)
Male-to-Female Ratio	2.6:1
Late Presentation	37 (46.3%)
Early Presentation	43 (53.8%)
Swelling Present	42 (52.5%)
Swelling Absent	38 (47.5%)
Non-availability of OT	16 (20%)
Availability of OT	64 (80%)

In our study, the mean length of hospital stay varied significantly between patients with late and early presentation across different subgroups. Overall, patients with late presentation had a longer mean hospital stay (3.04 ± 0.80 days) compared to those who presented early (2.36 ± 0.82 days), and this difference was statistically significant with a t-value of 4.103, indicating that delayed presentation was associated with a prolonged hospital stay.

When categorized by age, patients aged ≤ 50 years who presented late had a mean hospital stay of 2.98 ± 0.73 days while early presenters had a significantly shorter stay of 2.26 ± 0.81 days (t-value = 4.057). Similarly, for patients aged > 50 years, late presentation resulted in a mean stay of 3.18 ± 0.98 days compared to 2.50 ± 0.83 days for early presentation, with a t-value of 4.645, suggesting a notable difference in hospital stay between the age groups.

Gender-wise, late presentation in males was associated with a significantly longer hospital stay of 3.07 ± 0.83 days compared to 2.37 ± 0.82 days for early presentation (p = 0.038, t-value = 2.126). For female patients, late presentation led to a mean hospital stay of 2.95 ± 0.76 days while early presentation resulted in a stay of 2.33 ± 0.86 days, but the difference was not statistically significant (p = 0.119, t-value = 1.630).

Patients with swelling at the fracture site had a mean hospital stay of 3.02 ± 0.82 days for late presentation while early presenters stayed 2.73 ± 0.47 days. Although the p-value was 0.973, indicating no significant difference, the chi-square value of 0.0011 suggests a correlation between swelling and presentation timing. Patients without swelling showed a significant difference in hospital stay, with late presenters staying 3.17 ± 0.75 days and early presenters staying 2.23 ± 0.88 days (p = 0.239, chi-square = 0.0011).

The availability of the operation theater (OT) also influenced hospital stay. Patients with late presentation and non-availability of OT had a mean stay of 3.28 ± 0.75 days while early presenters stayed for 2.22 ± 0.67 days (p = 0.973, chi-square = 1.386). When the OT was available, late presenters had a mean hospital stay of 2.98 ± 0.81 days compared to 2.39 ± 0.86 days for early presentation (p = 0.239, chi-square = 1.386). This is presented in Table 3.

**Table 3:** Comparison of mean hospital stay between late and early presentation across different subgroups

Parameter	Late Presentation (Mean ± SD)	Early Presentation (Mean ± SD)	t-value	p-value	Chi-Square Value	F-Value
Overall Mean Hospital Stay (days)	3.04 ± 0.80	2.36 ± 0.82	4.103	-	-	16.84
Age ≤ 50 Years	2.98 ± 0.73	2.26 ± 0.81	4.057	-	-	16.45
Age > 50 Years	3.18 ± 0.98	2.50 ± 0.83	4.645	-	-	21.58
Male	3.07 ± 0.83	2.37 ± 0.82	2.126	0.038	-	4.52
Female	2.95 ± 0.76	2.33 ± 0.86	1.630	0.119	-	2.66
Swelling Present	3.02 ± 0.82	2.73 ± 0.47	-	0.973	0.0011	0.05
Swelling Absent	3.17 ± 0.75	2.23 ± 0.88	-	0.239	0.0011	0.07
Non-availability of OT	3.28 ± 0.75	2.22 ± 0.67	-	0.973	1.386	0.07
Availability of OT	2.98 ± 0.81	2.39 ± 0.86	-	0.239	1.386	0.07

## Discussion

Surgical management is the recommended approach for most unstable ankle fractures, as achieving precise anatomical reduction and minimizing damage to the joint surfaces are critical factors for favorable outcomes. Prompt reduction and stabilization of the soft tissues are essential for achieving optimal results, although immediate definitive fixation may not always be feasible. The timing of surgical intervention has been shown to affect bone healing, with delays of five to eight days, potentially leading to slower bone union at the six-week mark. This slower healing often requires prolonged periods of immobilization and non-weight-bearing, although the range of ankle motion at later follow-ups may remain unaffected [11].

In our study, while we did collect basic demographic and clinical data, comprehensive information on patient comorbidities, such as diabetes, hypertension, and cardiovascular diseases, was not systematically recorded or analyzed. Consequently, we did not perform a detailed assessment of how these comorbid conditions may have influenced the length of hospital stay. Patients who presented for surgery later had a mean hospital stay of 3.04 ± 0.80 days, compared to 2.36 ± 0.82 days for those who presented earlier, and this difference was statistically significant (p < 0.001). This suggests that surgical timing was a primary factor influencing hospitalization duration. The association between late presentation and longer hospital stays is consistent with findings from previous studies. For example, research has indicated that early surgery (within 24 hours) results in shorter average hospital stays of approximately 5.4 days, compared to 9.5 days for late surgery (beyond 24 hours), likely due to improved outcomes in physiotherapy and earlier mobilization [12]. A retrospective study involving 98 patients over a 14-month period similarly demonstrated that early surgery, occurring within 24 to 48 hours, was associated with significantly reduced costs and shorter hospital stays (p < 0.001) [13].

Interestingly, some studies have shown that the length of postoperative hospital stays may paradoxically be longer for early surgery (within 48 hours) compared to delayed surgery (6.98 vs. 4.93 days). This finding was attributed to variations in surgical thresholds and perioperative protocols, where patients with significant swelling or blistering were discharged and scheduled for semi-elective readmission once they were deemed ready for surgery [14].

Singh et al. reported that early surgery for ankle fractures significantly reduced postoperative hospital stays (2.9 vs. 5.5 days, p = 0.009). Their study highlighted the increased morbidity and longer hospital stays associated with delays in ORIF, advocating for surgery within 24 hours to minimize complications [15]. Similarly, Høiness and Strømsøe examined 84 patients with closed ankle fractures and found that surgical delays of five days or more increased the risk of soft tissue complications and extended hospital stays [9].

On the other hand, other research has not demonstrated a significant impact of early surgery on hospital stay duration, suggesting that additional factors, such as fracture type and perioperative management strategies, may play a significant role [16]. For instance, isolated fibular fractures, which account for approximately 70% of ankle fractures, can follow different clinical courses depending on their classification and treatment approach. Surgical intervention in these cases carries a 20% complication rate, emphasizing the need for careful patient selection and consideration of surgical timing [17,18].

Limitations

This study had several limitations, including a small sample size of 80 patients and its conduct at a single center, which may restrict the generalizability of the findings. The study primarily focused on short-term outcomes, such as the length of hospital stay, without assessing long-term effects, such as ankle mobility, pain, or quality of life, thus limiting the understanding of the full impact of surgical timing. Additionally, variability in postoperative care and discharge criteria, which were not standardized, could independently affect the length of hospital stays. Other potential confounding factors, such as patient comorbidities, fracture severity, and preoperative status, were not fully accounted for, and the reasons for surgical delays (e.g., swelling or operating theatre availability) were not systematically quantified, making it challenging to determine their exact contribution).

## Conclusions

This study underscores the importance of surgical timing in influencing the length of hospital stay for patients with closed ankle fractures. Patients who underwent early surgical intervention (within eight hours of injury) experienced shorter hospital stays compared to those whose surgery was delayed, highlighting the benefits of timely surgery in improving short-term outcomes. While factors such as swelling, operating theater availability, and patient-specific conditions can contribute to delays, addressing these issues proactively may help reduce hospital stays and associated healthcare costs. The study's findings suggest that early surgical management should be prioritized, when feasible, to enhance recovery and improve efficiency in patient care. However, further research involving larger, multicenter cohorts and standardized protocols is necessary to validate these results and explore the long-term impact of early surgery on functional outcomes and overall recovery.

## Figures and Tables

**Figure 1:**
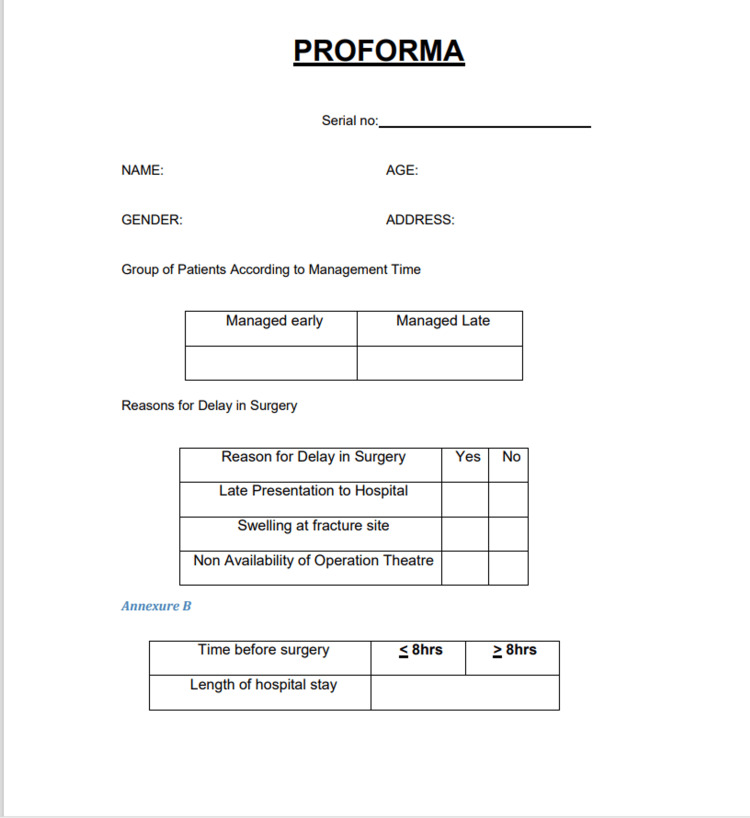
Proforma
